# Histomorphometric Study of the Tunics of Ductus Arteriosus in Human Fetal Cadavers Using the ImageJ Software

**DOI:** 10.7759/cureus.64148

**Published:** 2024-07-09

**Authors:** Meghna S Manohar, Balakrishnan Ramamoorthy, Ashma A Latiff, Sundarapandian Subramanian

**Affiliations:** 1 Department of Anatomy, Faculty of Medicine and Health Sciences, Sri Ramaswamy Memorial (SRM) Medical College Hospital and Research Centre, SRM Institute of Science and Technology, Kattankulathur, IND

**Keywords:** eosin and hematoxylin stains, image j software, ductus arteriosus, fetal cadavers, histomorphometry

## Abstract

Introduction: The ductus arteriosus (DA) connects the left pulmonary artery with the aorta during fetal life. Although it connects two elastic arteries, histological studies have shown that it is a muscular artery. There are very few studies on the histomorphometry of human fetal cadaveric DA. There are few studies on the changes in the tunics of the DA at various stages of fetal development. The present study aimed to observe the histomorphometric features of DA and its histological variations according to the gestational age of the fetus.

Methods: The study sample was DA dissected from 34 fetal cadavers of different gestational ages and stained with standard hematoxylin and eosin staining (H&E). We studied the structure of DA under a light microscope. We used ImageJ software (National Institutes of Health, Bethesda, Maryland) to measure the thickness of all three layers of the DA wall.

Results: The thickness of the DA wall was directly proportional to the gestational age of the fetus. In each trimester, we observed distinct histological changes in the tunics.

Conclusion: The formation of multiple intimal mounds and the increase in intimal thickness observed during the last trimester are responsible for the closure of the ductus after birth. Elastosis is associated with patent DA. The disappearance of elastosis at a later gestational age ensures the closure of the ductus.

## Introduction

The ductus arteriosus (DA) develops from the distal part of the left sixth aortic arch. It is a vascular structure connecting the proximal part of the descending aorta with the pulmonary trunk [1]. The DA shunts most of the deoxygenated fetal blood returning from the head, upper extremities, and coronary sinus through the right ventricle into the descending aorta because of the high vascular resistance of the collapsed fetal lung [2,3]. The diameter of the lumen of DA increases with an increase in the gestational week to reach the size equal to that part of the aorta with which it joins [4]. However, at birth, rapid changes in size and shape lead to constriction, and the closure of DA occurs. Although it directly connects two elastic arteries, the ductus is a muscular artery [5]. The DA has all three recognized layers of an artery: tunica intima (TI), tunica media (TM), and tunica adventitia (TA). The functional closure of DA occurs immediately after birth. In 90% of full-term babies, functional closure of the DA happens within 48 hours of birth. The structural closure of DA occurs due to the proliferation of the intimal cushion (IC) in TI. Permanent closure of DA occurs with complete obliteration in one to three months after birth.

The lack of histomorphometric analysis and histological correlation about the normal developmental stages of DA prompted the initiation of the current study. The current study aimed to observe and document the histomorphometry of all three tunics of DA in fetuses of all three trimesters and correlate it with the gestational age. It also highlights the microanatomy of DA at three levels: near the aorta, in the intermediate position, and near the pulmonary trunk.

## Materials and methods

A total of 34 aborted/stillborn fetuses were studied in the age group of 9-40 weeks (three to nine months) at the Department of Obstetrics and Gynecology, SRM Medical College Hospital and Research Centre, Kattankulathur, India. We embalmed the fetuses using cavity embalming, followed by surface treatment [6]. The embalming procedure was done in the Department of Anatomy, SRM Medical College Hospital and Research Centre, Kattankulathur, India.

Inclusion criteria

The study included spontaneously aborted fetuses from nine weeks to 40 weeks and stillborn fetuses.

Exclusion criteria

Fetuses with an identified congenital anomaly were excluded from the study.

Categorization of the fetuses

Based on the gestational age, we categorized the collected fetal samples into three groups: Group I: first trimester (9th-13th week), Group II: second trimester (14th-26th week), and Group III: third trimester (27th-40th week).

Dissection procedure

**Figure 1:**
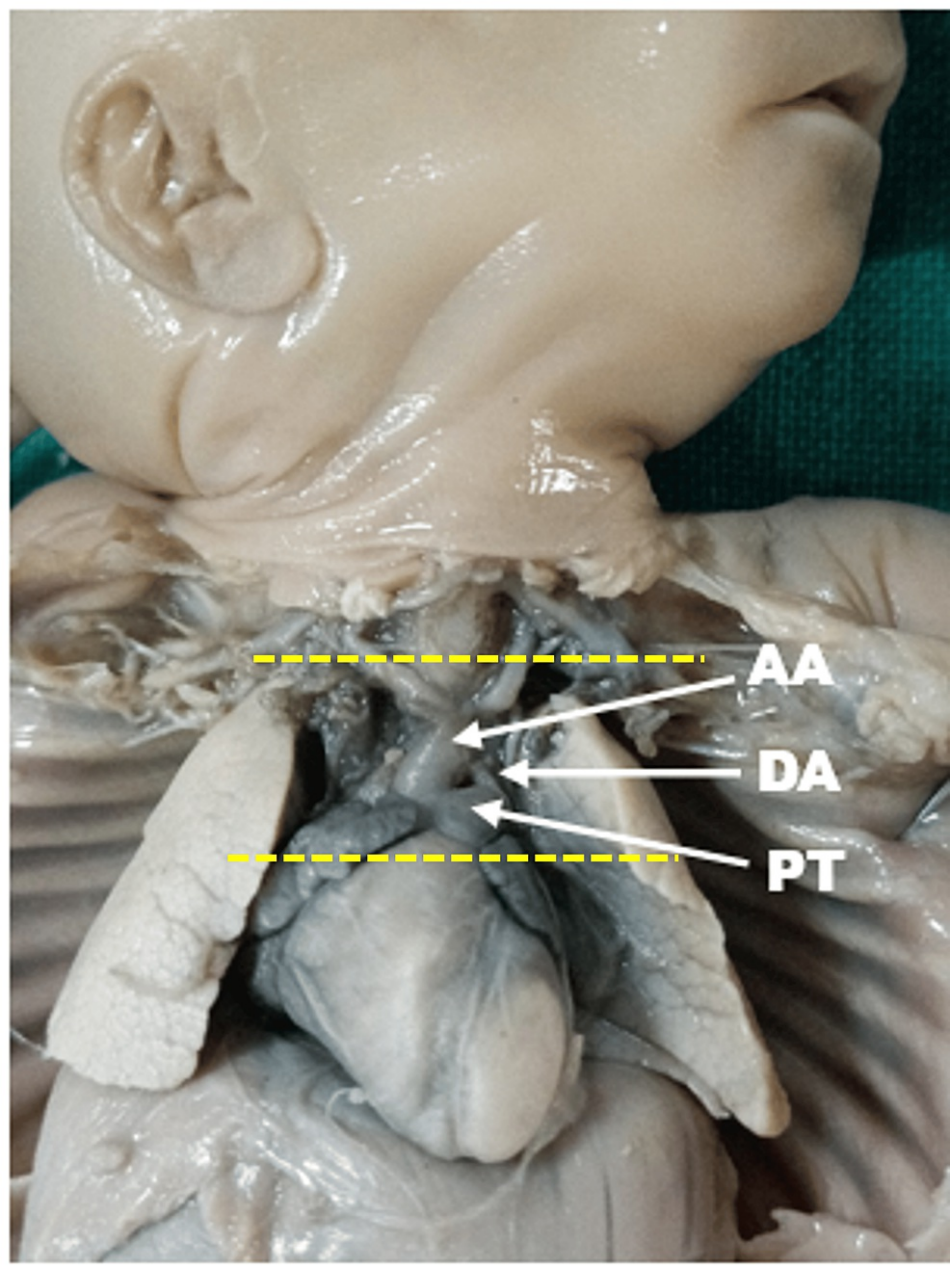
Dissection of the thoracic cavity of the fetus The two yellow lines represent the level of transverse incision made to remove the DA along with the AA and PT AA: arch of the aorta; DA: ductus arteriosus; PT: pulmonary trunk

We used an inverted Y-shaped incision from the jugular notch to the xiphoid process to dissect and open the thorax of the fetus. We then dissected the DA and the aorta and pulmonary roots by a transverse incision (Figure 1).

Histotechniques

We fixed the tissue in 10% formalin for 24 hours. After adequate fixation, we took three transverse sections from each sample. The first section was the DA near the pulmonary trunk, the second from the middle of the ductus, and the third near the aorta. Using standard procedure, we embedded the tissue in paraffin, serially sectioned at 5 microns, and stained with hematoxylin and eosin (H&E) [7].

Histomorphometric analysis

We observed the slides stained by the above process under a light microscope in low (40×), high (100×), and extremely high (400×) magnifications. We photographed the focused slides in all three magnifications through the eyepiece of the microscope using a mobile phone camera with Carl Zeiss lenses. We then uploaded the images in the ImageJ software (National Institutes of Health, Bethesda, Maryland) installed on a laptop. We measured and analyzed the thickness of each layer of the ductal wall using the software. The calibration was done using the ImageJ software's built-in "scale bar" tool. The measurements were taken in 100× magnification.

The measurements were taken at three points of maximal thickness, and their average was taken as the maximum thickness of that tunic. Similarly, three points of minimal thickness were measured, and their average was taken as the minimum thickness of the tunic. We measured the thickness of the intima, media, and adventitia at three different points of the vessel wall. The mean of the three values was the thickness of that tunic. The measurements were then correlated with the gestational age of the fetus and the histological findings in the respective age.

## Results

Histomorphometry

Table 1 presents the thickness of the three tunics of the DA in the three trimesters. The total thickness of the wall of the DA increased with gestational age. The thickness of each tunic also increased with gestational age. The thickness of the intima increased by about fourfold from the second trimester to the third trimester. This confirms the appearance of intimal mounds in the third trimester.

**Table 1:** Thickness of the ductal wall with respect to the gestational age of the fetus N: number of fetuses; µm: micrometers

Trimester	Weeks of Gestation	N	Thickness of DA Layers (µm)					
			Tunica intima		Tunica media		Tunica adventitia	
			Max	Min	Max	Min	Max	Min
I	8-13	3	3.71	1.86	25.40	18.14	23.61	12.53
II	13-26	28	9.91	03.40	77.97	19.10	69.7	14.58
III	26-40	3	37.67	10.80	79.81	28.33	16.79	9.37

The statistical analysis was carried out in IBM SPSS Statistics for Windows, Version 23 (Released 2015; IBM Corp., Armonk, New York). The Kolmogorv-Smirnov and Shapiro-Wilk tests were used to check for normality. The Kruskal-Wallis H test is a nonparametric rank-based test that was used to determine statistically significant differences in the thickness of TI, TM, and TA between the three trimesters. It is a nonparametric alternative to one-way ANOVA and an extension of the Mann-Whitney U test that allows comparison between more than two independent groups (Table 2).

**Table 2:** Mean thickness of the ductal wall with respect to the gestational age of the fetus N: number of fetuses; µm: micrometer

Tunic	Gestational Age	N	Mean Thickness (µm)
Tunica intima	1st trimester	3	2.00
	2nd trimester	28	17.61
	3rd trimester	3	32.00
Tunica media	1st trimester	3	3.00
	2nd trimester	28	18.86
	3rd trimester	3	19.33
Tunica adventitia	1st trimester	3	4.67
	2nd trimester	28	20.50
	3rd trimester	3	2.33

To evaluate the influence of the trimester on ductal layers, take the mean value of the thickness of the TI, TM, and TA for each gestational group. The test statistics table, which provides the result of the Kruskal-Wallis H test, can be used to determine whether there is an increase or decrease in the thickness of the TI, TM, and TA scores as the gestational age increases, i.e., the chi-squared statistic ("Chi-square" row), degrees of freedom ("df" row), statistical significance ("df" row), and the statistical significance of the test ("Asymp. sig." row) (Table 3).

**Table 3:** Test of significance a: Kruskal-Wallis test; b: grouping variable: gestational age

Test Statistics (a, b)			
	Tunica intima	Tunica media	Tunica adventitia
Chi-square	13.632	6.982	14.482
df	2	2	2
Asymp. sig.	.001	.030	.001

The Kruskal-Wallis H test showed that there was a statistically significant difference in the thickness of the TI, TM, and TA between the different trimesters: χ^2^(2) = 13.632, 6.982, and 14.482; p = 0.001, 0.030, and 0.001 with a mean rank score of TI in the first trimester (2), second trimester (17.61), and third trimester (32); TM first trimester (3), second trimester (18.86); and third trimester (19.33); TA first trimester (4.67), second trimester (20.5), and third trimester (2.33).

Group I: two to four months (9-13 weeks) of gestational age

**Figure 2:**
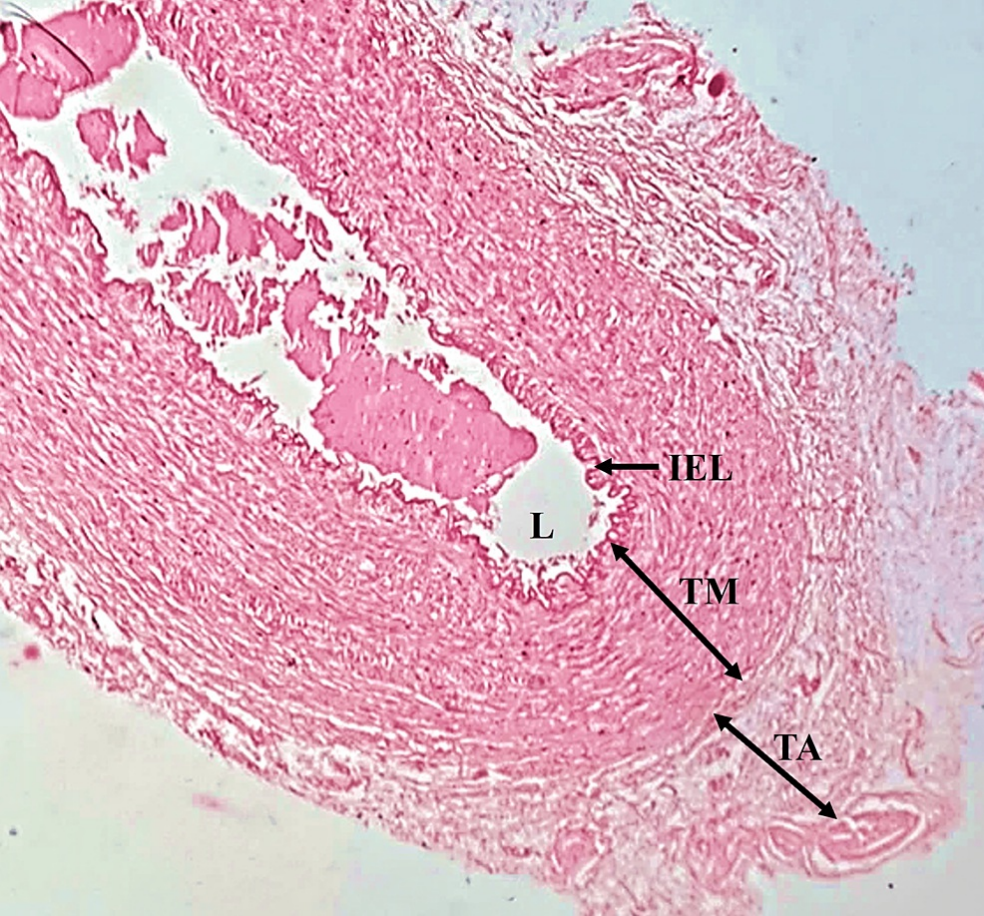
High magnification (400×) of DA from two to three months (8–13 weeks) of gestational age TM: tunica media; TA: tunica adventitia; L: lumen; IEL: internal elastic lamina

There were three fetuses in this group. In TI, the internal elastic lamina was very prominent. The absence of intimal cushion was the distinctive feature of this trimester. The TM consisted of many smooth muscle cells with few frail elastic fibers and was well demarcated from the other tunics. TA consisted of connective tissue and elastic fibers (Figure 2).

Group II: four to six months (14-26 weeks) of gestational age

**Figure 3:**
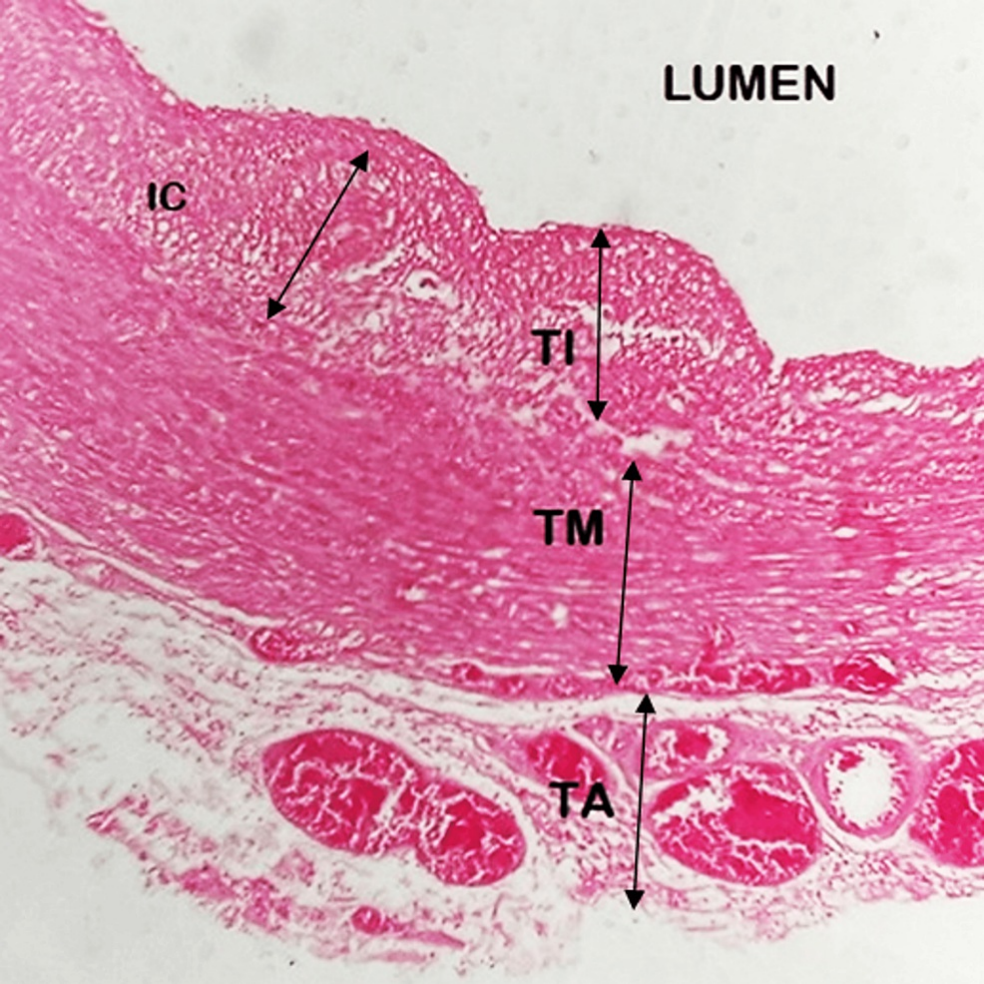
High magnification (400×) of the tunics of the ductus arteriosus at a gestational age of three to six months (14–26 weeks) TI: tunica intima; TM: tunica media; TA: tunica adventitia; IC: intimal cushion

A total of 28 fetuses belonged to this group. The DA of this group resembled a muscular artery. In the three to five months group, there were initial stages of the proliferation of TI. In the five to six months sections, we observed the histological features leading to ductal closure. Fragmentation of the internal elastic lamina occurred. Clear spaces appeared, and the beginning of the formation of intimal cushions or thickenings was noted. The intimal cushions were protruding toward the lumen in this stage. The TM showed predominant smooth muscle cells. The adventitia comprised connective tissue with abundant vasa vasorum (Figure 3).

Group III: six to nine months (27-40 weeks) of gestational age

In this group, the three layers of ductal walls were clearly visible. The TI increased in thickness with the presence of well-formed intimal cushions made of elastic and smooth muscle fibers protruding into the lumen. Intimal cushions consist of elastic fibers and smooth muscles arranged longitudinally and circularly in the TI. They appear in the third trimester. The disruption of continuity in the internal elastic lamina permits the migration of smooth muscle fibers and elastic fibers from the TM to the TI. This marks the beginning of the formation of intimal cushions. It is the precursor for the closure of DA after birth. Thus, the presence of smooth muscle cells in the TI is the definite criterion marking intimal mounds or cushions.

**Figure 4:**
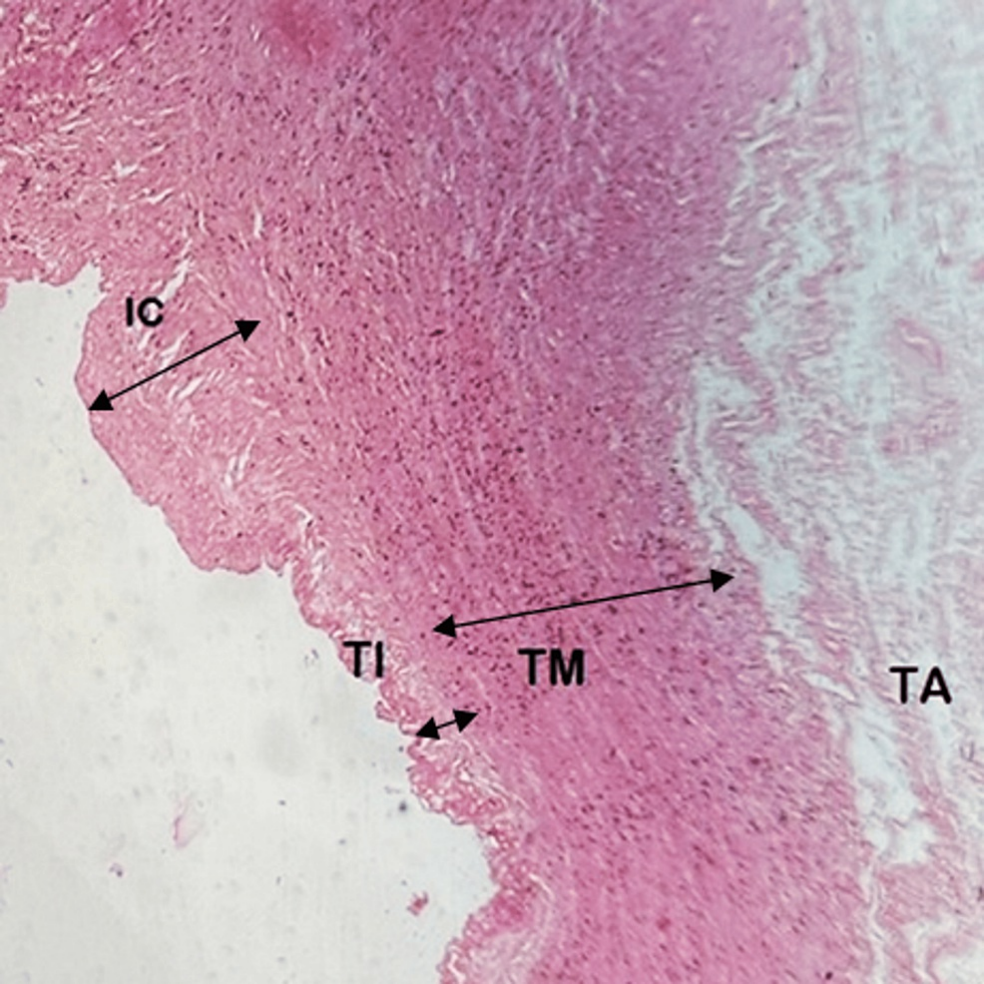
High magnification (400×) tunics of the ductus arteriosus at a gestational age of six to nine months (27–40 weeks) TI: tunica intima; TM: tunica media; TA: tunica adventitia; IC: intimal cushion

**Figure 5:**
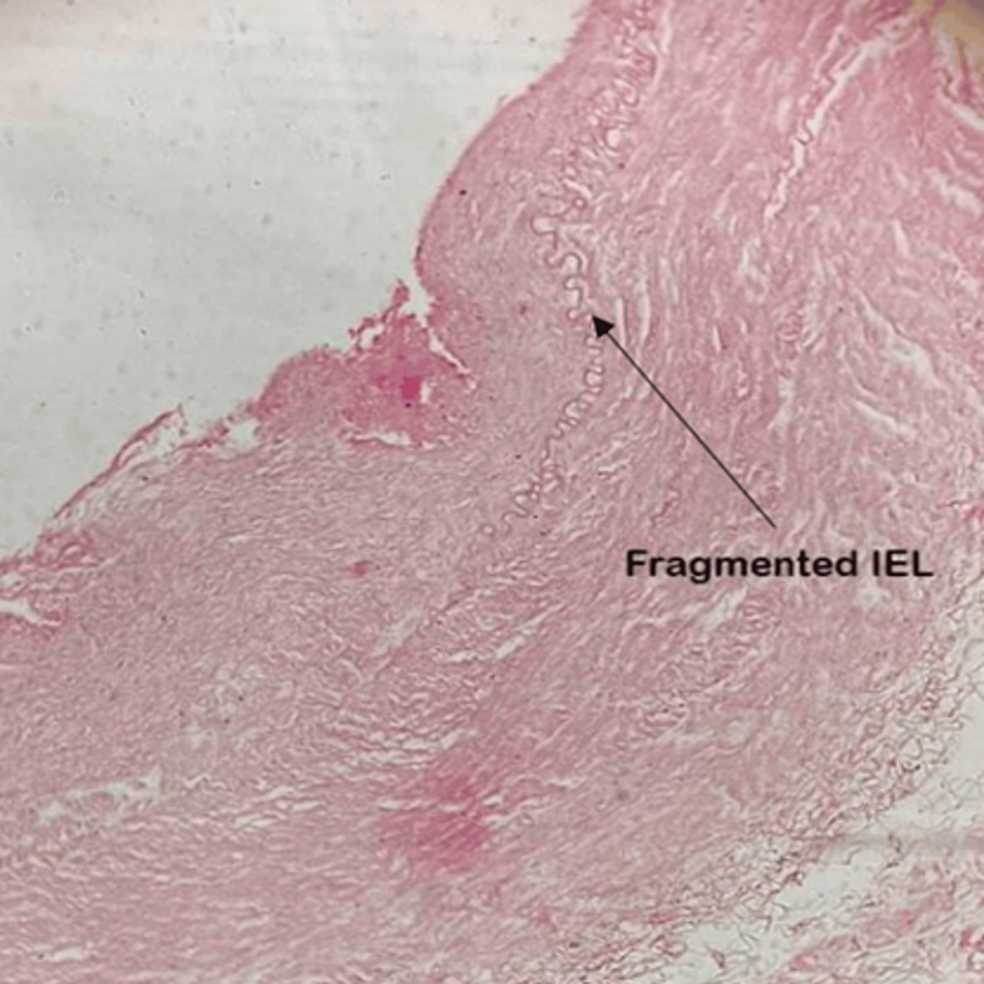
High magnification (400×) of the tunics of the ductus arteriosus at a gestational age of six to nine months (27–40 weeks). The fragmented internal elastic lamina (IEL) is visible IEL: internal elastic lamina

The fetuses aged six to seven months showed obliquely oriented smooth muscles migrating from the TM to the TI. In the full-term fetuses, intimal cushions were well-formed with the proliferation of subendothelial connective tissue. We observed disruption in the internal elastic lamina. Smooth muscle fibers were predominant in the TM. The TI showed a significant increase in thickness. No changes were noted in the TM's thickness in this group. The adventitia comprised connective tissue and vasa vasorum (Figures 4, 5).

Histology at the transitional zones (near the junction of DA with the pulmonary trunk and near the junction of DA with the aorta)

At the junction between the ductus and pulmonary trunk, the section resembled an elastic artery, with the TM comprising elastic fibers with very few smooth muscle cells. In the transitional zone between the ductus and the aorta, the TM of the ductus blended with the TM of the aorta. There were concentric lamellae of elastic fibers in the media. The histology of the transition zone was similar in all three groups, with no significant differences except for a decrease in elastic fibers in media nearing term.

Ductus elastosis

**Figure 6:**
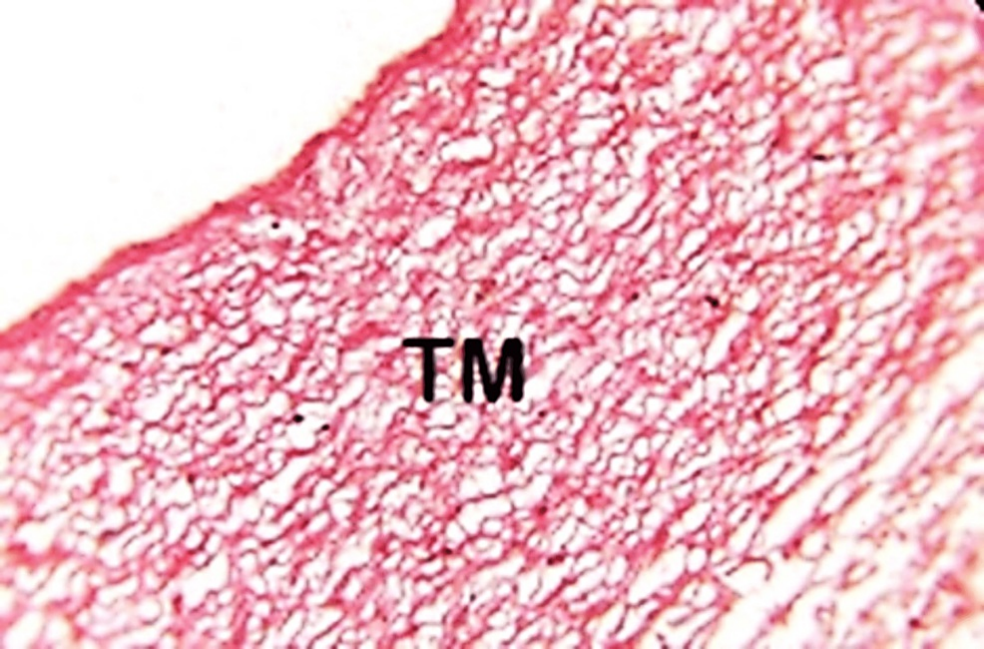
High magnification (400×) of the transitional zone of the ductus arteriosus at a gestational age of two to three months (8–13 weeks). The tunica media (TM) contains abundant elastic fibers TM: tunica media

A total of 12 out of 34 specimens had abnormally high amounts of elastic fibers in the TM. This is termed as ductus elastosis. Elastosis was seen in 11 fetuses in the second trimester and one fetus in the third trimester. The TM featured laminar elastosis or aortification, which is a primary characteristic associated with patent DA (Figure 6).

## Discussion

Microanatomy of DA

In the present study, the DA showed characteristics of a muscular artery that were different from those of the aorta and pulmonary trunk, which are elastic arteries. This matches the findings of Hughes [8], whose study aided in the classification of arteries developing from the aortic arches into three basic categories based on histological features: Type I and II belonging to elastic arteries and Type III as muscular arteries. The observation of DA being a muscular artery in the present study relates to the results of the study conducted by Yokoyama et al. [9]. Leonard et al. [10] observed that the left recurrent laryngeal branch of the vagus nerve enwrapping the DA as a sling-like support is responsible for the histological difference, allowing it to develop as a muscular artery in contrast to the arteries that develop from the adjacent unsupported aortic arches, which are elastic.

Tunica intima

Lining endothelium was visible without a prominent subendothelial connective tissue in the present study, which is similar to the observations of Jothi [11], in which the subendothelial connective tissue was barely visible. Desligneres and Larroche [5] also observed the appearance of intimal cushions in the second trimester. They also noted an increase in intimal thickness produced by "cushions" made of cells and metachromatic substances. These cushions were the thickening of media rather than intima. Meera et al. [12] noted a similar increase in the thickness of TI with disrupted internal elastic lamina. This discontinuity was due to the intimal smooth muscles showing connections with cells of the TM.

Tunica media

The TM of the DA consisted of an abundance of smooth muscle cells, according to Gournay [13], as in our present observation. In the present study, the TM in most samples was composed of circularly arranged smooth muscle fibers. However, in a few studies, there were outer longitudinal bundles of smooth muscles surrounded by connective tissue near the adventitia [11]. The TM is mainly comprised of smooth muscle cells and very few elastic fibers [5]. They also found clear spaces between the muscle fibers filled with metachromatic substances, which were absent in the present study. Costa et al. [4] observed well-defined external elastic lamina. However, in our observations, the external elastic lamina was either ill-defined or absent.

Tunica adventitia

In the present study, the TA comprises more of connective tissue elements, vasa vasorum and vasa nervosum. These observations are in accordance with the observations of Kuganathan et al. [12].

Transitional zones

the study conducted by Ågren et al. [14] on chicken DA had similar findings to the current study in the transitional zone. The presence of elastic fibers in the outer segment of the media and muscle fibers in the inner segment of the media was present in some samples. At the junction between the pulmonary artery and DA, the elastic fibers from the pulmonary artery continued to the external part of the media of DA on the side of the adventitia. The inner part of the media, near the intima, showed the typical features of the TM of the DA [5]. Danesino et al. [15] observed that the TM was composed of circularly arranged smooth muscle fibers and interspersed mucoid material. In another study, they measured the thickness of the three tunics [12] and noted an increase in the thickness of the wall of DA with the increase in the gestational age of the fetus, similar to the current study.

Closure of the ductus

Fetuses above six months of gestational age showed histological differences leading to DA obliteration. The observations of Yokoyama et al. [9] suggested that the obliteration of the DA after birth is significantly related to the decreased amounts of elastic fibers. Fetuses in early trimesters showed elastosis in the current study, while late trimester showed a reduction in elastic fibers. This supports the theory of patent DA being associated with elastosis. This was noted in the studies conducted by Toda et al. [16], Chaqui et al. [17], and Desligneres and Larroche [5].

Gournay [13] noted that in the media of the DA, there are abundant smooth muscle cells rather than the circumferentially oriented layers of elastic fibers that make up the media of the aorta and pulmonary artery. The lumen of the DA narrows, and the DA shortens due to the smooth muscle cell contraction present in the media, which leads to obliteration. The beginning of intimal thickening is seen as early as the fourth month of the second trimester with disruption of the internal elastic lamina in the present study, while in a similar study conducted by Desligneres and Larroche [5], the appearance of intimal cushions and the disruption of the internal elastic lamina is observed only after 32 weeks of gestation, which contributes to the closure of DA. The appearance of intimal mounds as a focal thickening on one side of the wall in the early months of the second trimester and the formation of multiple mounds in the later stages are similar to the observations made by Kuganathan et al. [12]. The migration of smooth muscle fibers from the TM to the TI marked the initiation of obliteration of DA. The oblique direction of muscle fibers entering the intima disrupted the internal elastic lamina. This caused fragmentation of internal elastic lamina in our study, as well as in another study [18].

Limitations

In this study, we primarily relied on H&E staining. Using special stains such as Masson’s trichrome or Verhoeff’s stain for collagen and elastic fibers, respectively, could provide additional insights.

## Conclusions

In this study on the histomorphometry of DA in fetal cadavers, the thickness of each layer of DA was directly proportional to the gestational age of the fetus. Each trimester showed distinct histological changes. The first appearance of intimal mounds was in the second trimester as a focal thickening at any one side of the wall. The formation of intercellular spaces, migration of smooth muscles from media to intima, and disruption of internal elastic lamina occurred gradually with an increase in the gestational age of fetuses. The formation of multiple mounds and the increase in intimal thickness observed during the last trimester mark the onset of the obliteration process of DA.
